# Mal de Río Cuarto virus infection causes hormone imbalance and sugar accumulation in wheat leaves

**DOI:** 10.1186/s12870-019-1709-y

**Published:** 2019-03-22

**Authors:** Luis Alejandro de Haro, Sofía Maité Arellano, Ondrej Novák, Regina Feil, Analía Delina Dumón, María Fernanda Mattio, Danuše Tarkowská, Gabriela Llauger, Miroslav Strnad, John Edward Lunn, Stephen Pearce, Carlos María Figueroa, Mariana del Vas

**Affiliations:** 1 Instituto de Biotecnología, CICVyA, INTA, CONICET, Hurlingham, Buenos Aires Argentina; 2 Laboratory of Growth Regulators, Palacký University and Institute of Experimental Botany Czech Academy of Sciences, Šlechtitelů 27, CZ-78371 Olomouc, Czech Republic; 3 Max Planck Institute of Molecular Plant Physiology, Potsdam-Golm, Germany; 4 Instituto de Patología Vegetal, CIAP, INTA, Córdoba, Argentina; 5 Department of Soil and Crop Sciences, Colorado State University, Fort Collins, CO USA; 6 Instituto de Agrobiotecnología del Litoral, UNL, CONICET, FBCB, Santa Fe, Argentina

**Keywords:** Disease symptoms, *Fijivirus*, MRCV, Plant hormones, *Reoviridae*, Sucrose metabolism, SWEET transporters, Trehalose 6-phosphate, Wheat

## Abstract

**Background:**

Mal de Río Cuarto virus (MRCV) infects several monocotyledonous species including maize and wheat. Infected plants show shortened internodes, partial sterility, increased tillering and reduced root length. To better understand the molecular basis of the plant-virus interactions leading to these symptoms, we combined RNA sequencing with metabolite and hormone measurements.

**Results:**

More than 3000 differentially accumulated transcripts (DATs) were detected in MRCV-infected wheat plants at 21 days post inoculation compared to mock-inoculated plants. Infected plants exhibited decreased levels of *TaSWEET13* transcripts, which are involved in sucrose phloem loading. Soluble sugars, starch, trehalose 6-phosphate (Tre6P), and organic and amino acids were all higher in MRCV-infected plants. In addition, several transcripts related to plant hormone metabolism, transport and signalling were increased upon MRCV infection. Transcripts coding for GA20ox, D14, MAX2 and SMAX1-like proteins involved in gibberellin biosynthesis and strigolactone signalling, were reduced. Transcripts involved in jasmonic acid, ethylene and brassinosteroid biosynthesis, perception and signalling and in auxin transport were also altered. Hormone measurements showed that jasmonic acid, brassinosteroids, abscisic acid and indole-3-acetic acid were significantly higher in infected leaves.

**Conclusions:**

Our results indicate that MRCV causes a profound hormonal imbalance that, together with alterations in sugar partitioning, could account for the symptoms observed in MRCV-infected plants.

**Electronic supplementary material:**

The online version of this article (10.1186/s12870-019-1709-y) contains supplementary material, which is available to authorized users.

## Background

*Mal de Río Cuarto virus* (MRCV, genus *Fijivirus*, family *Reoviridae*) causes the most important viral maize disease in Argentina [1], the world’s second largest maize exporter (https://atlas.media.mit.edu/en/visualize/tree_map/hs92/export/show/all/1005/2016/). Other viruses in this genus cause severe rice and maize diseases in Asia [2, 3]. Besides maize, MRCV infects several monocotyledonous crops such as wheat (*Triticum aestivum*), oat (*Avena sativa*), rye (*Secale cereale*) and diverse species of weeds that act as winter reservoirs [4, 5]. The virus is transmitted by a number of species of delphacid planthoppers in a persistent propagative manner [6–9]. Viral replication is phloem-limited and causes severe symptoms in plants, whereas replication in planthoppers is asymptomatic [10].

Its high infection rate [10], the growing and handling advantages over maize and the availability of genetic and genomic resources [11–13] makes wheat an excellent model species to study Mal de Río Cuarto disease [14].

One of the earliest disease symptoms in wheat (appearing around 20 days post inoculation, dpi) is a darker coloration in basal leaves. Then, leaves become shorter, erect and coriaceous. Curled leaves with cross-cut edges and thickened ribs are commonly observed (Additional file 1: Figure S1). Sporadically, hypertrophy of the phloem leads to the presence of white waxy tumors called enations that are located along the veins [4, 5]. In addition, infected plants exhibit severe shortening of internodes and roots, a stunted appearance and an increased number of tillers in the vegetative stage. In the reproductive stage, plants show deformed ears that are partially or totally sterile [5]. Interestingly, symptoms occasionally appear only in a single tiller [4].

Viral infection triggers a cascade of events that profoundly disrupt host physiology, leading to virus-induced disease symptoms [15–17]. This process is systemically coordinated by a fine-tuned system in which phytohormones play a master role. Moreover, viral symptoms often resemble the phenotypes of mutants with compromised hormone biosynthesis, signalling or transport [18]. In general, salicylic acid (SA), cytokinins (CKs) and brassinosteroids (BRs) exert positive effects on plant defence against viruses, whereas auxins, ethylene (ET) and jasmonic acid (JA) have negative effects. While abscisic acid (ABA) can have both positive and negative effects in response to bacteria and fungi, it predominantly enhances plant defences upon virus infections [19].

Sugar availability is one of the major determinants of plant growth and is influenced by a large number of abiotic and biotic external factors. The impact of biotic interactions on sugar availability has been extensively studied for bacteria and fungi that colonize the apoplast, but much less is known regarding the impact of virus infections that exclusively replicate in the symplast [20–22]. Plant viral infections were reported to alter carbohydrate allocation and signalling. Soluble sugars and starch accumulate in the leaves where the virus is actively replicating, photosynthesis is reduced and respiration increased [20, 23]. The underlying mechanisms are specific to each plant-virus pathosystem and can involve the expression of viral movement proteins that interfere with plasmodesmata function and/or callose deposition, thus altering their function [24–26].

The vast majority of studies on the molecular basis of plant-virus interactions were conducted in eudicotyledonous plants, such as *Arabidopsis thaliana* (hereafter Arabidopsis) and *Nicotiana benthamiana*. Far less is known about the physiological responses of grasses to viral infections [16]. In the present study we performed an exhaustive analysis of the transcriptome, metabolite and hormone profiles of systemic MRCV-infected wheat leaves at two early time points of infection. We found that MRCV infection causes a strong alteration in sugar partitioning, characterized by high levels of sucrose, starch and the signal metabolite Tre6P. At the transcriptomic level, we found 3233 DATs at 21 dpi, including reduced transcript levels of nine genes encoding sucrose transporters from the SWEET (Sugars Will Eventually be Exported Transporters) family and for class II Tre6P synthases (TPS). In addition, our results implicated several phytohormones in the production of viral symptoms.

## Results

### Effect of MRCV-infection on the wheat transcriptome

Controlled infection experiments were performed to compare the wheat transcriptome between MRCV-infected and mock-inoculated plants. We were particularly interested in understanding the changes that may contribute to the establishment of a systemic infection rather than the late, pleiotropic effects of the infection. Therefore, the youngest fully expanded systemic leaf of each plant was sampled at 12 and 21 dpi. Plants at 12 dpi are asymptomatic, whereas the latter time point coincides with the appearance of macroscopic symptoms (Additional file 1: Figure S1). For each time point, four infected plants with similar viral loads and four mock-inoculated plants treated with non-viruliferous planthoppers were selected. We extracted total RNA from the individual leaf samples, constructed RNA-seq libraries and sequenced them using Illumina technology. On average, 50.0 million reads per library were obtained, 98.2% of which were retained after adapter and quality trimming (Additional file 1: Figure S2). Reads were mapped to the wheat genome (assembly version TGACv1) and, using only uniquely mapped reads (~ 43% of the total), we identified DATs between infected and control plants at each time point. At 12 dpi, before the appearance of disease symptoms, we identified just two DATs (FDR-adjusted *P*-value< 0.05, Additional file 2: Table S1). This result indicates that MRCV infection has almost no impact on the wheat transcriptome at this early time point. In contrast, at 21 dpi, following the onset of visual disease symptoms, we identified 3233 DATs, 42% with lower and 58% with higher transcript levels in infected plants compared to mock-inoculated samples (Additional file 2: Table S1). Twelve DATs at 21 dpi were randomly selected to validate the RNA-seq by qRT-PCR. We found consistent results using both technologies (Student’s t Test, *P* < 0.05), indicating that RNA-seq data is reliable (Additional file 1: Figure S3).

**Fig. 1:**
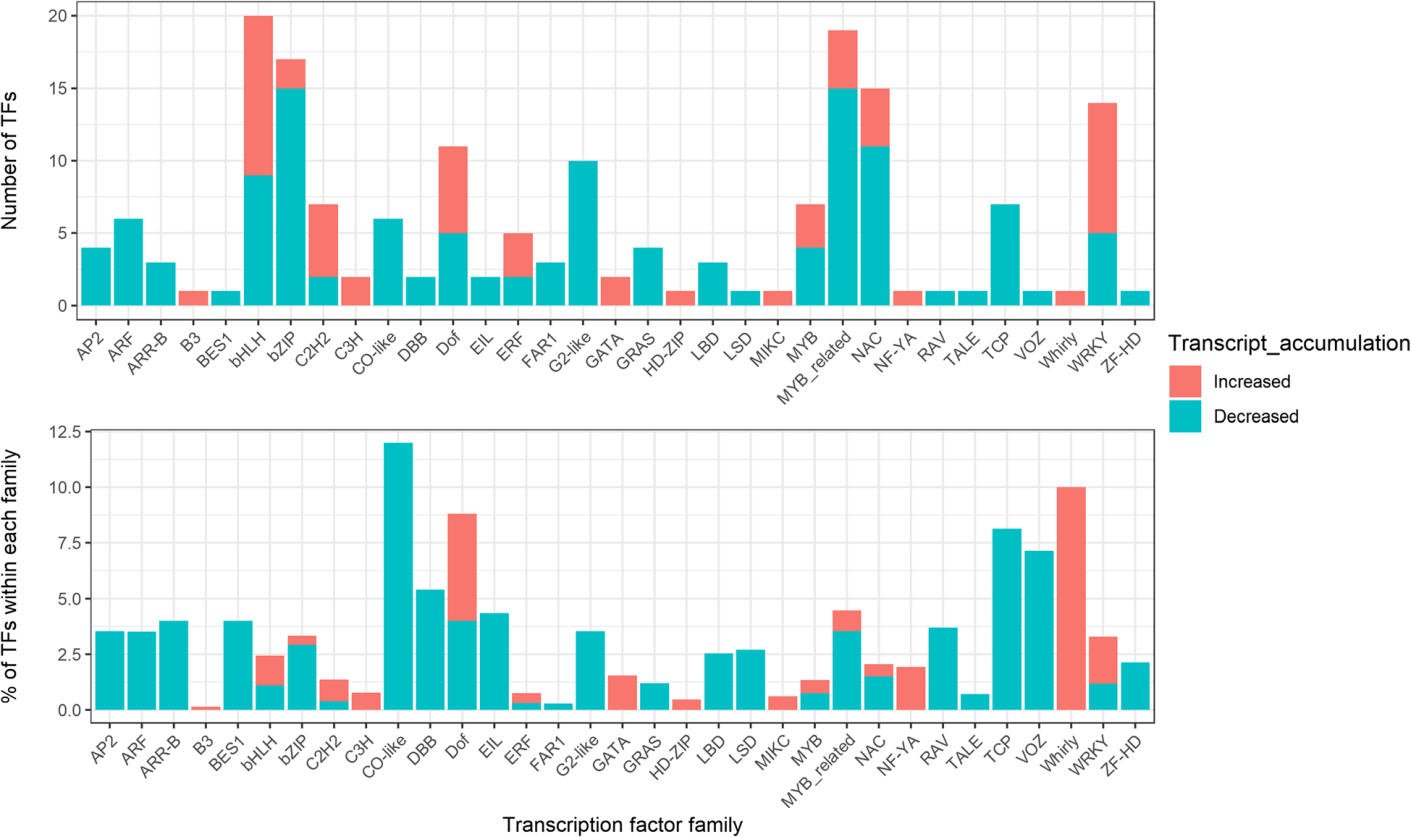
Effect of MRCV infection on transcripts coding for TFs of different families. Absolute number (upper panel) and proportion within each family (lower panel) of DATs coding for TF upon the infection. Red and blue colours represent increased and decreased transcripts, respectively

Using a set of annotated wheat transcription factors (TFs) [27], we identified 180 TFs from 33 different families among DATs at 21 dpi (Fig. 1 and Additional file 2: Table S2). Although 58% of the total DATs showed increased accumulation in infected plants, 68.9% of the transcription factor DATs showed reduced levels upon the infection. TFs from the basic helix-loop-helix (bHLH), MYB-related, basic leucine zipper (bZIP), NAC and WRKY families were the most represented. When analyzing the proportion of altered TFs within each family, CONSTANS-like (CO-like), WHIRLY, DNA BINDING WITH ONE FINGER (Dof), TEOSINTE-BRANCHED1/CYCLOIDEA/PCF (TCP) and VASCULAR PLANT ONE-ZINC FINGER (VOZ) families were most affected by MRCV infection (Fig. 1). Some of the TFs found to be altered upon MRCV infection are involved in hormone responses and are discussed below.

We next performed a gene ontology (GO) analysis to identify classes of enriched genes within DATs at 21 dpi (Table 1). Over-represented GO categories for the 1874 DATs with higher levels in infected plants included translation, cellulose biosynthesis and oxylipin biosynthetic process (Table 1). Among the 1359 DATs with reduced levels in infected plants, enriched GO categories included carbohydrate transport, trehalose biosynthesis, CK biosynthesis and strigolactone (SL) signalling.

**Table 1:** Over-represented GO categories of DATs during infection for “Biological Process” ontology (Fisher’s exact test with *P* < 0.05)

GO ID	Term	Annotated	Significant	Expected	classicFisher
GO categories of increased DATs					
GO:0006412	Translation	1624	257	42.09	< 1e-30
GO:0030244	Cellulose biosynthetic process	117	22	3.03	3.4e-13
GO:0006414	Translational elongation	121	20	3.14	4.7e-11
GO:0034975	Protein folding in endoplasmic reticulum	6	6	0.16	3.0e-10
GO:0007017	Microtubule-based process	366	30	9.49	1.1e-08
GO:0006457	Protein folding	346	42	8.97	3.6e-08
GO:0007010	Cytoskeleton organization	322	21	8.35	5.3e-08
GO:0042026	Protein refolding	32	9	0.83	8.4e-08
GO:0030245	Cellulose catabolic process	60	11	1.56	3.6e-07
GO:0031408	Oxylipin biosynthetic process	47	9	1.22	2.9e-06
GO:0006564	L-serine biosynthetic process	17	6	0.44	2.9e-06
GO:0006165	Nucleoside diphosphate phosphorylation	186	12	4.82	1.9e-05
GO:0006183	GTP biosynthetic process	14	5	0.36	1.9e-05
GO:0006228	UTP biosynthetic process	14	5	0.36	1.9e-05
GO:0006167	AMP biosynthetic process	14	4	0.36	6.8e-05
GO:0042254	Ribosome biogenesis	269	22	6.97	7.8e-05
GO:0006166	Purine ribonucleoside salvage	5	3	0.13	0.00017
GO:0006555	Methionine metabolic process	46	8	1.19	0.00032
GO:0000028	Ribosomal small subunit assembly	6	3	0.16	0.00033
GO categories of decreased DATs					
GO:0045454	Cell redox homeostasis	379	37	5.96	1.4e-18
GO:0005992	Trehalose biosynthetic process	56	11	0.88	1.1e-09
GO:0019538	Protein metabolic process	11,517	136	181.11	1.1e-07
GO:0006809	Nitric oxide biosynthetic process	9	5	0.14	1.1e-07
GO:1904143	Positive regulation of carotenoid biosynthesis	9	5	0.14	1.1e-07
GO:0048577	Negative regulation of short-day photoperiodism, flowering	9	5	0.14	1.1e-07
GO:0015798	Myo-inositol transport	6	4	0.09	8.9e-07
GO:0009691	Cytokinin biosynthetic process	37	7	0.58	1.6e-06
GO:1902348	Cellular response to strigolactone	3	3	0.05	3.9e-06
GO:0042128	Nitrate assimilation	17	5	0.27	5.0e-06
GO:0016573	Histone acetylation	61	8	0.96	5.1e-06
GO:0008643	Carbohydrate transport	151	12	2.37	5.3e-06
GO:0008654	Phospholipid biosynthetic process	148	9	2.33	2.2e-05
GO:1901562	Response to paraquat	5	3	0.08	3.8e-05
GO:0019375	Galactolipid biosynthetic process	5	3	0.08	3.8e-05
GO:0071492	Cellular response to UV-A	6	3	0.09	7.5e-05
GO:0006021	Inositol biosynthetic process	6	3	0.09	7.5e-05
GO:0009585	Red, far-red light phototransduction	17	4	0.27	0.00012
GO:0009584	Detection of visible light	8	3	0.13	0.00020
GO:0006821	Chloride transport	35	5	0.55	0.00021

### Effect of MRCV infection on primary metabolism and sugar allocation

Viral replication demands the biosynthesis of a great amount of new molecules in infected tissues and thus has a strong impact on primary metabolism [23]. At the transcript level, we identified 34 DATs involved in carbohydrate synthesis, degradation and transport, according to their associated GO terms and Ensembl annotations (Additional file 2: Table S3). Four sucrose synthase transcripts were significantly increased, including four transcripts with homology to Arabidopsis sucrose synthase 5 and 1. In addition, 12 transcripts coding for invertases and/or fructosyltransferases (evolutionarily related to vacuolar invertases) were altered at 21 dpi (Additional file 2: Table S3). Vacuolar invertases and fructosyltransferases directly affect the levels of soluble sugars and may thus contribute to the generation of turgor, which further drives cell expansion [28]. Since wheat invertases and fructosyltransferases are not fully annotated in the TGACv1 assembly version of the wheat genome, we built a phylogenetic tree including sequences of previously reported homologs to better classify them [29, 30]. We identified four significantly decreased DATs coding for vacuolar invertases and eight significantly increased DATs encoding fructosyltransferases (1 sucrose:sucrose 1-fructosyltransferase, 1-SST; 4 fructan:fructan 1-fructosyltransferases, 1-FFT; and 3 sucrose:fructan 6-fructosyltransferases, 6-SFT). In line with a possible accumulation of fructans in the infected plants, a fructan 6-exohydrolase involved in fructan degradation was decreased upon the infection (Additional file 1: Figure S4, Additional file 2: Table S3). In addition, 12 transcripts encoding homologs of enzymes involved in starch metabolism (2 glucoamylases, 2 granule-bound starch synthases, 2 branching enzymes, and 6 alpha-glucan phosphorylases) were significantly increased, while four β-amylase transcripts were significantly decreased and one was increased at 21 dpi (Additional file 2: Table S3). We also found evidence of alterations in cell wall remodeling transcripts. The GO categories of cellulose biosynthetic and catabolic processes were over-represented among transcripts with increased levels at 21 dpi (Table 1). These categories include several cellulose synthase, endoglucanase and xyloglucan endotransglucosylase/hydrolase transcripts (Additional file 2: Table S3).

**Fig. 2:**
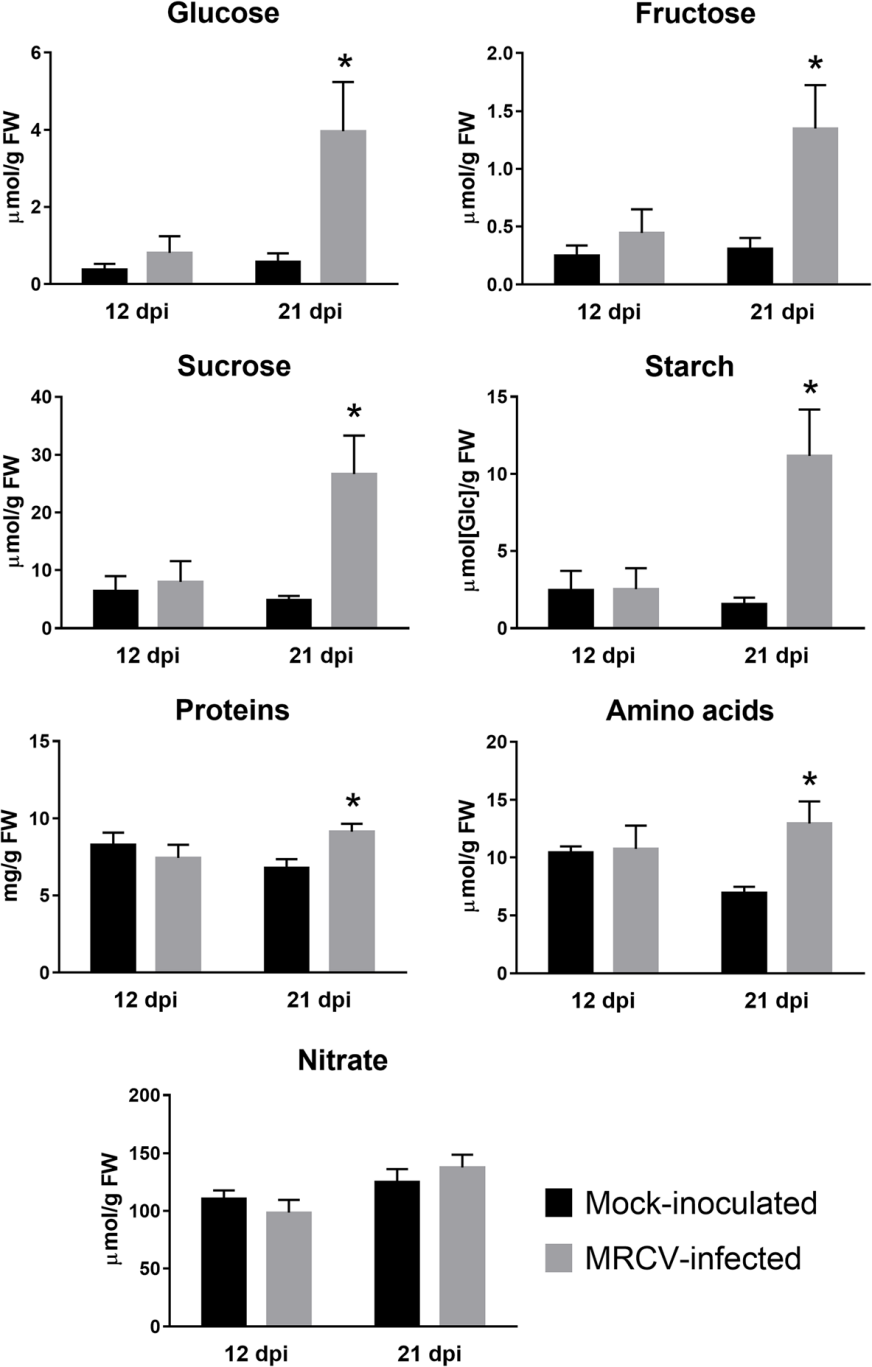
Effect of MRCV infection on the accumulation of sugars, starch, total amino acids, proteins and nitrate in MRCV-infected wheat leaves at 12 and 21 dpi. Student’s t test: *P < 0.05; *n* = 5. Error bars: standard error

**Fig. 3:**
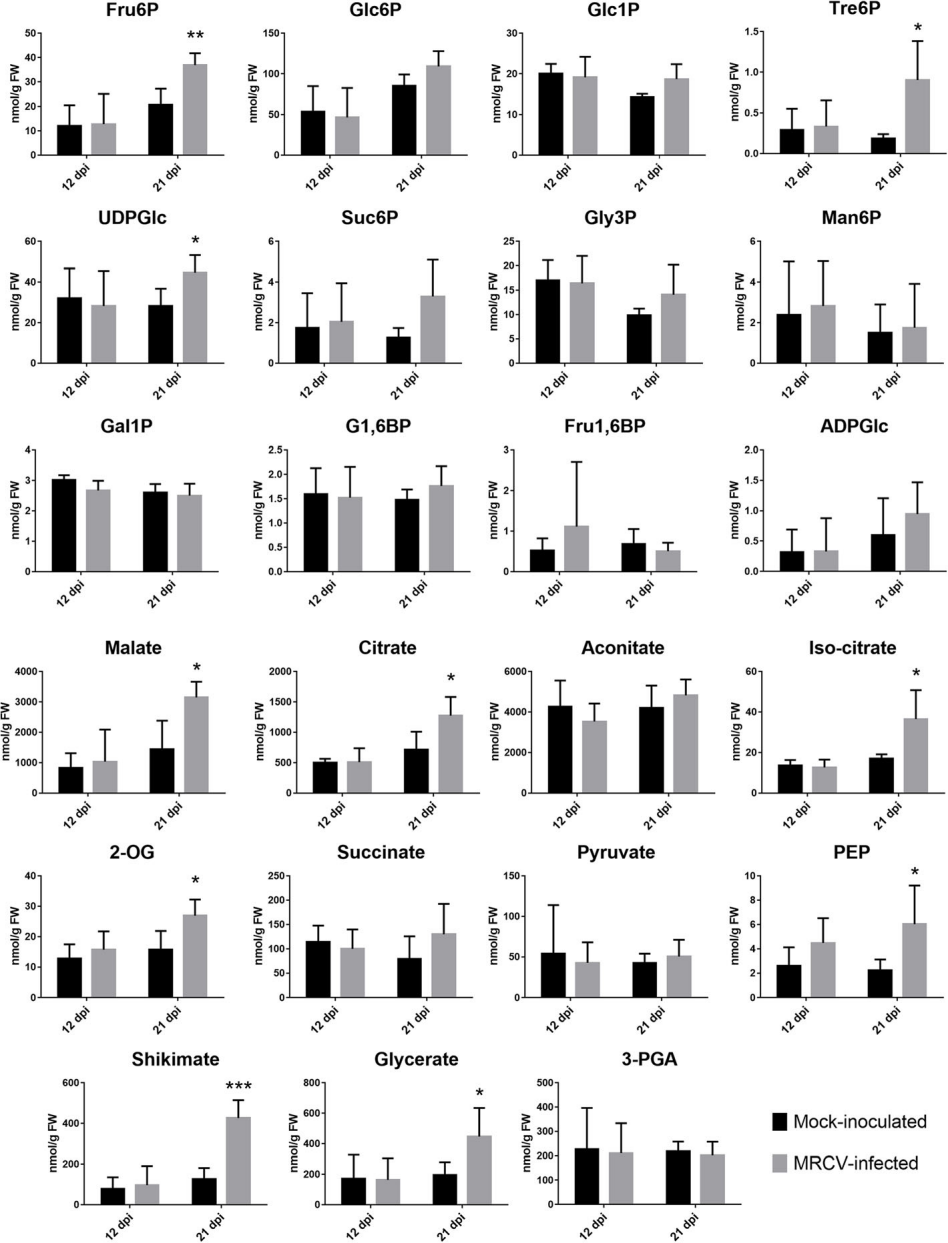
Accumulation of primary metabolites during MRCV infection at 12 and 21 dpi. Metabolites were analyzed by LC-MS/MS. Student’s t test: *P < 0.05, **P < 0.01, ***P < 0.001; *n* = 5. Error bars: standard error

The changes observed in transcripts coding for enzymes involved in primary metabolism prompted us to quantify soluble sugars and starch. MRCV-infected plants showed a significant increase in glucose, fructose, sucrose and starch at 21 dpi (Fig. 2, *P* < 0.05). In agreement with the gene expression analysis, we found no significant differences for any of these metabolites between MRCV-infected and control plants at 12 dpi. To further investigate the metabolic status of MRCV-infected plants, we used liquid chromatography coupled to tandem mass spectrometry (LC-MS/MS) to determine the levels of phosphorylated intermediates and organic acids. No differences were observed for any of these metabolites at 12 dpi; conversely, 10 out of 23 metabolites were significantly accumulated in MRCV-infected leaves at 21 dpi (Fig. 3, *P* < 0.05).

Interestingly, the signal metabolite Tre6P was 5-fold more abundant in MRCV-infected leaves at 21 dpi than in controls (Fig. 3). Tre6P is both a signal and a negative regulator of sucrose levels in plants, thus linking growth and development to carbon status [31, 32]. In illuminated Arabidopsis leaves, Tre6P promotes the synthesis of organic and amino acids by post-translational activation of phosphoenolpyruvate (PEP) carboxylase and nitrate reductase [33]. Similarly, the levels of Tre6P and certain amino acids concomitantly increased in axillary buds from decapitated pea plants [34]. In line with these findings, we observed accumulation of several tricarboxylic acid (TCA) cycle intermediates (citrate, iso-citrate, 2-oxoglutarate and malate), PEP, shikimate and total amino acids in MRCV-infected plants at 21 dpi (Figs. 2 and 3). Total protein levels were also increased, whereas nitrate content was not significantly impacted by MRCV infection (Fig. 2).

The GO category trehalose biosynthesis was over-represented among transcripts with decreased levels at 21 dpi (Table 1). Previous phylogenetic analyses of plant TPS proteins showed that they cluster in two distinct sub-families [35, 36]. Class I TPS proteins are catalytically active, whereas the function of class II TPS proteins remains unknown [32, 37, 38]. Because wheat *TPS* genes are not fully annotated in the TGACv1 assembly version of the wheat genome, we built a phylogenetic tree including 21 putative wheat TPS proteins (Additional file 2: Table S4) and previously reported TPS sequences from Arabidopsis, rice, poplar and common bean [35, 36, 39]. Additional file 1: Figure S5 shows that 11 DATs coding for class II TPSs (TaTPS2, TaTPS5, TaTPS6 and TaTPS10) were significantly decreased, whereas two transcripts encoding TaTPS4 (another class II TPS) were increased.

The GO category carbohydrate transport was over-represented among DATs with decreased levels at 21 dpi (Table 1). We considered this interesting since it has been shown that bacterial and fungal plant pathogens are able to hijack sucrose transporters [22, 40, 41]. In Arabidopsis leaves, sucrose efflux from phloem parenchyma cells to the apoplast is driven by AtSWEET11 and 12 [42, 43]. Interestingly, we found 13 altered *SWEET* transcripts in MRCV-infected leaves at 21 dpi (Additional file 2: Table S3). To further characterize these transcripts, we searched for sequences coding for SWEET transporters in the wheat genome and found 90 putative members for this family (Additional file 2: Table S4). Next, we built a phylogenetic tree including sequences from Arabidopsis and rice [40, 44]. The levels of nine transcripts coding for *TaSWEET13* were decreased in MRCV-infected plants. The proteins encoded by these transcripts cluster with AtSWEET11/12 and OsSWEET14 (Additional file 1: Figure S6), which are sucrose exporters located in phloem parenchyma cells [42].

Amino acid metabolism is also modulated by viral infections [23]. Interestingly, we observed a significant accumulation of amino acids and total proteins upon MRCV infection (Fig. 2, *P* < 0.05). The levels of several transcripts encoding enzymes involved in amino acid synthesis were altered at 21 dpi (22 increased and 6 decreased; Additional file 2: Table S5). The recent identification of wheat amino acid transporters [45] allowed us to detect 18 transcripts significantly altered upon MRCV infection (15 decreased and 3 increased; Additional file 2: Table S5), suggesting that amino acid transport is also affected by MRCV infection. Overall, our results show that MRCV infection has a profound effect on carbon partitioning and amino acid metabolism.

### Effect of MRCV infection on phytohormone metabolism and transport

**Fig. 4:**
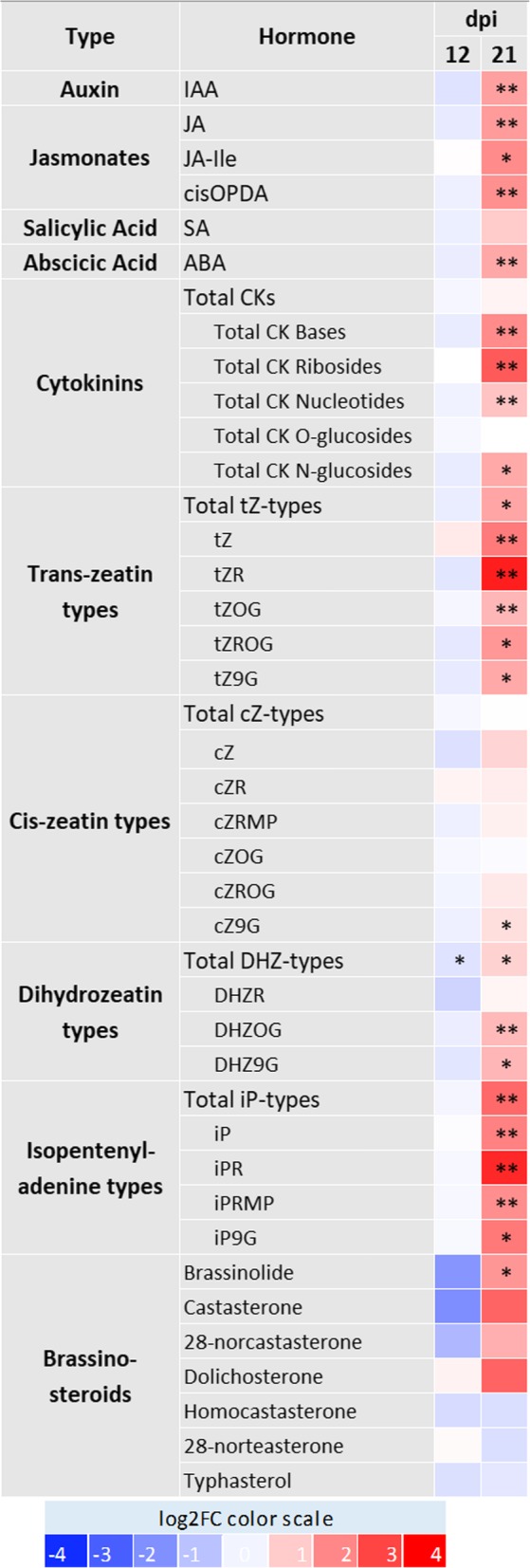
Heat-map visualization of hormone accumulation in MRCV-infected plants at 12 and 21 dpi. The log_2_FC color-scale is indicated. Asterisks indicate statistically significant difference between infected and mock-inoculated plants. Student’s t tests; * and ** correspond to *P* < 0.01 and *P* < 0.001, respectively; *n* = 5

Changes in hormone metabolism, transport and signalling are frequently associated with virus symptoms. Thus, we analyzed transcripts involved in phytohormone synthesis, catabolism, transport and signalling that were significantly altered in MRCV-infected plants at 21 dpi. We also quantified several hormones including their biosynthetic precursors and metabolites by ultra-high performance liquid chromatography–electrospray tandem mass spectrometry (UHPLC–MS/MS). The transcripts associated with the different hormones are listed and classified in Additional file 2: Table S6, while Fig. 4 shows the fold change of hormone levels between infected and mock-inoculated plants. Each hormone is discussed separately in the sections below.

#### Cytokinins

The levels of eight transcripts of *LOG* (*LONELY GUY*) genes were reduced in MRCV-infected plants. These genes encode cytokinin riboside 5′-monophosphate phosphoribohydrolase, an enzyme that converts inactive cytokinin nucleotides to the biologically active free-base forms [46]. In addition, transcript levels of three type-B ARR (AUTHENTIC RESPONSE REGULATOR) family members (Fig. 1), which are associated with cytokinin signal transduction [47], were also decreased. These results suggest that MRCV infection may be associated with a reduction in CK activity. However, when we measured different CK forms, total CK bases (bioactive forms), CK ribosides (transport forms), CK nucleotides (precursor forms) and CK N-glucosides (irreversible catabolites) were all significantly increased upon MRCV infection.

#### Brassinosteroids

A wheat transcript homologous to Arabidopsis and rice *DIMINUTO/DWARF1* (*DIM*/*DWF1*) genes was significantly more abundant in MRCV-infected plants. These proteins are involved in the synthesis of the BR precursor campesterol [48, 49]. By contrast, *DWF5*, a gene encoding a protein involved in BR biosynthesis [50] was significantly decreased in MRCV-infected plants. Remarkably, the abundance of a transcript encoding the BR receptor BRASSINOSTEROID INSENSITIVE1 (BRI1) [51] was 19-fold higher in infected plants than in controls. Finally, a transcript coding for the transcription factor BES1/BZR1 and three transcripts coding for 14-3-3-like proteins identified as BZR1- and BZR2/BES1-interacting proteins [52] were significantly reduced in MRCV-infected plants. These transcriptional changes are consistent with a reduction in the levels of bioactive BRs. However, when we measured the endogenous levels of these compounds, the most active form of BRs, brassinolide (BL), was 3.4 times more abundant in MRCV-infected plants (Fig. 4).

#### Auxin

WALLS ARE THIN1 (WAT1) is a vacuolar auxin transport facilitator required for auxin homeostasis [53]. Six *WAT1* transcripts were decreased while eight transcripts were significantly increased upon infection. In addition, three transcripts coding for the F-box TRANSPORT INHIBITOR RESPONSE 1 (TIR1) auxin receptor [54], two transcripts coding for the auxin carrier PIN5 (PIN-FORMED5) [55] and two others coding for PIN-LIKES [56] were decreased. Moreover, the levels of six transcripts coding for auxin response factors (ARFs, Fig. 1) [57] were reduced. Consistent with these expression profiles, we found that the levels of IAA, the most abundant of the four native forms of auxins, were 3-fold higher in MRCV-infected leaves (Fig. 4).

#### Abscisic acid

The levels of an abscisic aldehyde oxidase *AAO2* transcript involved in ABA and AUX biosynthesis [58] rose upon MRCV infection, while the levels of an ABA 8′-hydroxylase transcript involved in ABA catabolism [59] were reduced. In addition, seven transcripts coding for ABA receptors PYL1 and PYL5 [60], were decreased and three transcripts coding for SKP1-like protein involved in ABA signalling [61] and the levels of TANDEM ZINC FINGER PROTEIN 1 (TZF1) (Fig. 1), which confers hypersensitivity to ABA seedlings in rice [62], were increased. Consistent with these expression profiles, we found that the levels of ABA were approximately three times higher in infected plants (*P* < 0.001) (Fig. 4).

#### Ethylene

The levels of a transcript coding for 1-aminocyclopropane-1-carboxylate synthase (ACS), which catalyses the generally rate-limiting step in ethylene biosynthesis [63], and two transcripts coding for ethylene-responsive transcription factors [64] were 40-, 6.5- and 2.2-fold higher in infected plants, respectively.

#### Jasmonic acid

The levels of 5 transcripts that code for lipoxygenase enzymes LOX2 involved in JA biosynthesis [65] were elevated in MRCV-infected plants. In addition, 14 WRKY TFs, some of them known to be induced by MeJA treatment in wheat and rice [66], showed altered expression in MRCV-infected plants (Fig. 1). The levels of transcripts coding for JA-responsive proteins involved in defence such as pathogenesis-related and germin-like proteins were elevated (Additional file 2: Table S1). We found that the endogenous levels of jasmonates were approximately 3.5-fold higher in MRCV-infected plants than control plants (Fig. 4).

#### Salicylic acid

SA synthesis depends on the activity of methylesterases [67]. The levels of three transcripts coding for methylesterases were increased while the levels of one of these transcripts was decreased upon infection. However, the levels of SA were not significantly affected by MRCV infection (Fig. 4).

#### Gibberellin

Bioactive GA levels strongly depend on 2-oxoglutarate-dependent dioxygenases (2-ODDs: GA20ox, GA3ox, GA2ox), which catalyze the latter steps of the biosynthetic pathway [68]. Transcript levels of two homeologous copies of *GA20ox2* (*GA20ox-A2* and *GA20ox-D2*), were decreased. These expression profiles are consistent with MRCV-infection being associated with a reduction in GA biosynthesis. In addition, two transcripts coding for gibberellin receptor GID1 were increased upon the infection [69].

#### Strigolactones

SLs are hormones that inhibit tillering and shoot branching through the MORE AXILLARY BRANCHING (MAX)-dependent pathway [70–72]. Remarkably, in MRCV-infected plants we found a significant decrease in transcript levels of the genes encoding the putative SL receptor strigolactone esterase D14 [73, 74]; the second component of the pathway, F-box/LRR-repeat MAX2 from Arabidopsis [75] and rice [76]; and SMAX1-LIKE proteins with homology to Arabidopsis SMXL 6, 7 and 8, which act redundantly in response to SL [77, 78].

Collectively, these results indicate that MRCV infection causes a profound disruption in hormone accumulation. Some of these changes could be linked to variations in the levels of transcripts coding for transcription factors and proteins responsible for hormone biosynthesis, transport or degradation.

## Discussion

Grasses are the primary source of calories for humans and livestock [79]. Understanding the molecular basis underlying their growth, development and defence is particularly relevant for the foundation of breeding strategies for increasing and stabilizing yields. As plant viruses induce profound disruptions in host development and physiology, it will be important to dissect these mechanisms to identify key elements that can aid the improvement of agricultural traits.

To study MRCV infection in wheat, we chose two time points that precede (12 dpi) or coincide with (21 dpi) the appearance of macroscopic symptoms. We found only 2 DATs and no significant changes in metabolite levels in MRCV-infected plants at 12 dpi, whereas we detected large-scale alterations at 21 dpi (Figs. 2 and 3 and Additional file 2: Table S1). Importantly, MRCV non-structural proteins P5–2, P7–1 and P7–2 are localized in the nucleus in insect and plant cells [80, 81] where they could affect the regulation of host target genes, as has been shown for rice black streaked dwarf virus (RBSDV) P8 non-structural protein [82].

We found that both sucrose and Tre6P accumulated in fully expanded wheat leaves during MRCV infection at 21 dpi (Figs. 2 and 3). Sucrose accumulation in leaves has been repeatedly reported after virus infections [83, 84]. Soluble sugars and starch accumulate in MRCV-infected wheat leaves during late stages of infection [85], while several proteins involved in carbohydrate metabolism and photosynthesis were differentially accumulated in maize leaf tumours caused by RBSDV [86]. However, to the best of our knowledge, Tre6P accumulation has never been reported in virus-infected plants. According to the Tre6P-sucrose nexus model, the Tre6P:sucrose ratio is part of a homeostatic mechanism that ensures the maintenance of sucrose levels within an appropriate range for each cell type and/or developmental stage [31]. We observed that Tre6P and sucrose proportionally increased in MRCV-infected plants at 21 dpi compared to controls (Figs. 2 and 3); i.e. the Tre6P:sucrose ratio was not affected by the viral infection (0.040 and 0.037 nmol μmol^− 1^ for infected and control plants, respectively). These values are similar to those reported for Arabidopsis rosettes and maize leaves [32], suggesting that the Tre6P-sucrose nexus is still functional in these plants. In Arabidopsis, Tre6P regulates starch turnover [87, 88] and coordinates organic and amino acid metabolism with carbon availability [33]. Accordingly, we observed accumulation of starch, organic acids (including several TCA intermediates) and total amino acids in infected plants at 21 dpi (Figs. 2 and 3). Accumulation of Tre6P has been linked to reproductive failure [89, 90]; interestingly, seed abortion is one of MRCV symptoms [5]. We speculate that reduced sucrose export from source tissues due to MRCV infection is perceived as sugar starvation by developing spikes, thus leading to seed abortion.

We found 13 class II *TPS* transcripts with altered levels in MRCV-infected plants at 21 dpi (Additional file 1: Figure S5 and Additional file 2: Tables S3 and S4). From these, 11 DATs were decreased (*TaTPS2*, *TaTPS5*, *TaTPS6* and *TaTPS10*) and two DATs were increased (*TaTPS4*) upon MRCV infection. This is consistent with previous reports showing divergent transcriptional regulation of different *TPS* genes [31]. Transcripts coding for TaTPS2 and TaTPS6 cluster with AtTPS7, while those encoding TaTPS4 and TaTPS10 are in the same branch as AtTPS6 (Additional file 1: Figure S5). These results are consistent with those observed for *AtTPS5–7* transcripts in C-starved Arabidopsis seedlings 3 h after sucrose re-addition; i.e. high Tre6P and sucrose levels and concomitant, opposite changes in *AtTPS5* and *AtTPS6–7* transcripts [31]. It has been suggested that all Arabidopsis class II TPS (AtTPS5–11) lack catalytic activity, as they fail to complement the *tps1Δ* and *tps1Δtps2Δ* yeast mutants [37, 38]. As the functions of the class II TPSs are largely unknown, it is unclear whether the increased Tre6P levels are linked to the changes in class II TPS expression. One possible hypothesis is that Tre6P and *TPS* transcript levels reflect the high sucrose content of these plants [31]. Nevertheless, class II TPS proteins could play a key role in sugar sensing and Tre6P homeostasis [91, 92]. The use of novel reverse genetics resources for wheat [11, 93] will be invaluable to study the specific functions of the different members of this clade.

It has previously been shown that transcripts coding for the sucrose transporters AtSWEET11 and 12 are induced by fungal and bacterial pathogens that colonize the apoplast. As a consequence, sugar efflux is increased at the site of infection, affecting both pathogen growth and plant immunity [22, 94]. We observed a significant decrease in the levels of nine paralogous and homeologous *TaSWEET13* transcripts and a concomitant increase of *TaSWEET15* transcripts in MRCV-infected plants at 21 dpi (Additional file 1: Figure S6 and Additional file 2: Table S4). Likewise, the homologous genes in rice [95], sorghum [96] and Arabidopsis [97] showed opposed expression patterns in different physiological situations. We speculate that these changes may lead to reduced sucrose phloem loading, which in turn would contribute to the observed sucrose accumulation in source leaves (Fig. 2).

Interestingly, maize SWEET13 paralogs (a, b and c) are highly expressed in the leaf vasculature and phloem loading is impaired in the triple *zmsweet13a, b, c* knock-out mutant [98]. In MRCV-infected wheat, impaired sucrose phloem loading could reduce carbon partitioning to the roots (and other sink tissues), negatively affecting their growth, which is a well-known symptom of MRCV. Strikingly, AtSWEET13 and 14 mediate cellular GA uptake when expressed in yeast and *Xenopus* oocytes and defects of double *atsweet13/14* mutants (such as delayed anther dehiscence) are reversed by GA treatment [99]. It is tempting to speculate that reduction in *TaSWEET13* levels caused by MRCV infection could modulate GA responses, thus contributing to disease symptoms in wheat. It would be interesting to characterize the phenotype of wheat *sweet13* loss-of-function mutants to test this hypothesis.

High sucrose levels in MRCV-infected leaves might have multiple consequences. From a plant physiology perspective, it could downregulate photosynthesis and promote starch and fructan accumulation [100–102]. In addition, the increase of the cell osmotic potential caused by sucrose accumulation could be partially relieved by cell wall expansion and/or by a decrease in the activity or expression of vacuolar invertases. Consistent with this hypothesis, we identified 39 DATs involved in cell wall synthesis, remodeling and expansion and 4 transcripts coding for vacuolar invertases with reduced accumulation at 21 dpi.

From a viral life-cycle perspective, high sucrose levels in infected tissues could contribute to increased carbon availability to sustain viral replication [23] and to attract planthopper vectors for feeding on infected plants or modify their feeding behaviors. Interestingly, non-viruliferous planthoppers prefer fijivirus-infected rice plants than healthy ones, whereas persistently-infected planthoppers prefer to feed on healthy plants [103]. The attractiveness of fijivirus-infected rice plants to planthoppers is related to virus titer and varies along the virus infection cycle [104].

Sugar, hormone signalling and defence networks are profoundly intertwined since hormone-driven processes need to be energetically compatible with the carbon status of the plant. Numerous mechanistic links between sugar and hormone-mediated processes have been described, and were recently reviewed. Moreover, several studies connect sucrose with auxin production and transport [105]. Accordingly, we detected high levels of auxin along with a reduction of transcripts coding for ARFs and for PIN5, PIN-likes and WATs auxin transporters along with high sucrose levels in MRCV-infected leaves (Additional file 2: Table S6).

Dwarfism is the most conspicuous symptom caused by fijivirus infection. It was recently shown that RBSDV P7–2 non-structural protein binds both rice S-PHASE KINASE-ASSOCIATED PROTEIN 1 (SKP1) and GIBBERLLIN INSENSITIVE DWARF2 (GID2). Since GID2 is essential for regulating the GA signalling pathway [106], these results suggest that GA signalling may play an important role during fijivirus infection [107]. In wheat, we found that *TaGA20ox-A2* and *–D2* transcript levels were significantly decreased in MRCV-infected plants (Additional file 2: Table S6). *GA20ox2* encodes a protein which catalyzes the rate-limiting step during GA biosynthesis [108]. In rice, *OsGA20ox2* loss-of-function mutations are responsible for the semi-dwarf phenotype of “green revolution” varieties [109] and in barley, reduced expression of *HvGA20ox2* is also associated with a semi-dwarf phenotype [110]. We hypothesize that decreases in *GA20ox2* transcript levels contribute to the dwarfed phenotype characteristic of MRCV infection in wheat. It will be important to assay bioactive GA levels in MRCV-infected plants to test this hypothesis.

We also found that the levels of transcripts encoding key SL signalling components, such as D14, MAX2 and SMAX1-like proteins SMXL 6, 7 and 8, were significantly reduced in MRCV-infected plants (Additional file 2: Table S6). In Arabidopsis, a MAX2 homolog is regulated by sugar, and both MAX2 and SMXL 6, 7 and 8 proteins promote auxin transport in the stem and growth of leaf length and width [77]. Although we were unable to quantify SL levels in our samples, the key role of these components suggests that MRCV infection may partially impair SL signalling causing excessive tillering and contributing to the reduced root and altered leaf morphology observed in MRCV-infected plants. The participation of SL in fijivirus symptoms has not been previously proposed.

Intriguingly, MRCV infection decreased the transcript levels of several LOGs involved in CK synthesis and ARR transcription factors involved in CK signaling. Yet, CK bioactive, transport and precursor forms were increased upon infection. This is in agreement with the result that *ahk* loss-of-function mutants have increased cytokinin content [111] and thus underpins the existence of homeostatic control mechanisms. It was further supported by analysis of endogenous cytokinin concentrations in the *rock2* and *rock3* mutants with enhanced cytokinin signalling. Taken together, the gain-of-function alleles of *AHK2* and *AHK3* have an impact on cytokinin homeostasis and lower the cytokinin content supporting a feedback regulation of cytokinin metabolism by the cytokinin signalling pathway [112]. Part of these control mechanisms is an influence of cytokinin signalling on the transcript level of cytokinin metabolism genes [113].

MRCV infection increased both bioactive BRs and transcript levels of the BR receptor *BRI1* (Fig. 4 and Additional file 2: Table S6). Interestingly, *TaBRI1* overexpression in Arabidopsis increased BR sensitivity and resulted in root length inhibition in a concentration-dependent manner [114]. Therefore, enhanced BR signalling could contribute to the reduced root length phenotype caused by MRCV infection.

Finally, MRCV-infected plants also exhibited increased ABA content. The antiviral roles of ABA were recently reviewed [19]. In other viral infections, ABA accumulation increases callose deposition on plasmodesmata, thus limiting viral spread [19, 115]. Interestingly, two *CalS* transcripts with homology to *OsCal7* and *AtCal7* that are responsible for specific callose deposition in the phloem [116] were significantly accumulated.

## Conclusions

Fijiviruses cause severe rice and maize diseases that threaten crop production worldwide [1–3, 117] while wheat and other winter species act as virus reservoirs. Our work provides novel insights into the mechanisms underlying fijivirus symptom development in wheat highlighting for the first time the participation of the signal metabolite Tre6P, *TaSWEET13* transcripts and the SL and GA signaling pathways in this process. The recent establishment of CRISPR/Cas9 genome editing protocols [11, 118, 119], along with the availability of sequenced TILLING populations both in tetraploid and hexaploid wheat [11, 93], will contribute to further deepen into these mechanisms.

## Methods

### Plant inoculation and RNA sequencing

Six-day-old wheat (*T. aestivum* cv. ProINTA Federal) seedlings were infected with MRCV as previously described [14]. This cultivar was selected because it is susceptible to MRCV infection [4]. Briefly, second instar *Delphacodes kuscheli* (delphacid planthopper vector) nymphs fed on MRCV-infected wheat plants were used for 1:1 transmission assays. Nymphs fed on non-infected wheat were used as control treatment. Plants were grown in 750 cm^3^ pots with soil sterilized by solarization in greenhouses with temperature-controlled conditions (24 ± 3 °C) in 16h light / 8h dark photoperiod and daily irrigation with no fertilization. The plants were rotated regularly within the greenhouse to reduce any positional effects. The youngest fully-expanded leaf was collected at 12 and 21 dpi, frozen in liquid nitrogen and kept at − 80 °C until use. Wheat plants were identified as MRCV infected or non-infected by DAS-ELISA at 50 dpi [4]. Absolute viral load was quantified as previously described [14, 120] and plants with similar viral load (log_10_(MRCV-S3 molecules)/(μg of total RNA) = 20.60 ± 0.96) for each time point were selected. To avoid leaf oxidative stress caused by ovipositions, only plants infected by male delphacids were used. Total RNA samples from mock-inoculated and MRCV-infected plants were extracted at 12 and 21 dpi (*n* = 4, a total of 16 individual samples) using a mirVana kit (Thermo Fisher Scientific Inc., Waltham, MA, USA) following the total RNA extraction protocol. cDNA libraries were synthesized using an Illumina TruSeq RNA sample prep kit (Illumina, San Diego, CA, USA). Library quality was checked using a High Sensitivity DNA chip in a Bioanalyzer 2100 kit (Agilent Technologies, Santa Clara, CA, USA). Libraries were sequenced on the HiSeq 3000 platform (Illumina, San Diego, CA, USA) at the UC Davis Genome Center using a single-end 50 bp (SE 50) module. Raw sequences of the 16 libraries were deposited in NCBI Sequence Read Archive (SRA) under the accession SRP160433.

### Sequence data analysis

Raw reads were processed as previously described [121]. Briefly, “Scythe” (https://github.com/vsbuffalo/scythe) was used to remove adapter contamination (default options) and “Sickle” (https://github.com/najoshi/sickle) to remove low-quality reads (default options except –l 25 –q 25). Trimmed reads were mapped to the Chinese Spring (CS42) TGACv1 genome assembly [12] using GSNAPl (version 2017-09-11, default parameters except -m 2 -n 1 -N 1 -A sam) [122]. Raw counts for gene features were obtained using ht-seq count (default parameters except -m union -a 30) [123] using the GFF file available in Ensembl plants release 37. A threshold for mapping quality was selected so that only uniquely-mapped reads were retained, ensuring homoeologue-specific expression profiles [124]. DESeq2 version 1.17.39 [125] and R version 3.4.0 were used for the normalization and classification of differentially accumulated transcripts (DATs) between infected and mock-inoculated samples (FDR-adjusted *P*-value ≤0.05, Additional file 2: Table S1).

Annotation data were retrieved from Ensembl plants release 37 using BioMart [126, 127]. GO annotation was used for functional enrichment analysis using the ‘R’ package TopGO version 2.29.0. “Biological Process” ontology was used and the significance values were calculated using ‘classic’ Fishers’ exact test (*P* < 0.01). When detailed annotation data were not available for *T. aestivum*, data from the closest homolog of *Aegilops tauschii* (goatgrass, the diploid progenitor of the D-genome of hexaploid wheat) was used. A reference-quality genome sequence was recently reported with more than 76% of genes annotated [128].

### qRT-PCR validations

One μg of DNase-treated total RNA, SuperScript III Reverse transcriptase (Invitrogen, Carlsbad, CA, USA) and random primers were used to synthesize the first-strand of cDNA, according to the manufacturer’s instructions. Twelve DATs were randomly selected and the respective oligonucleotides (listed in Additional file 2: Table S7) were designed using Primer3 [129]. SYBR green PCR reactions were carried out in 20 μl final volume containing: 3 mM MgCl_2_, 200 nM each primer, 0.2 mM dNTPs, 1 U Platinum *Taq* DNA Polymerase (Invitrogen, Carlsbad, CA, USA), 20 mM Tris-HCl (pH 8.4), 50 mM KCl. Cycling conditions were as follows: 95 °C for 5 min, 40 cycles at 95 °C for 15 s, 60 °C for 60 s and a final extension at 72 °C for 30 s, in an Applied Biosystems Step One Plus Real Time PCR system. qRT-PCR data analysis and primer efficiencies were obtained using LinRegPCR software [130]. The genes coding for *GTP-BINDING PROTEIN* (*GTPB*, Genbank ID: JQ673330.1) and *ELONGATION FACTOR 1 ALPHA* (*EF1α*, Genbank ID: AK455225.1) were used as reference [131]. Relative expression ratios and statistical analysis were performed using fgStatistics software interface [132]. Six biological replicates (the same four as in the RNA-seq plus two additional replicates) and two technical replicates were performed. Statistical significance between RNA-seq and qPCR Fold Change values was assessed by Two-tailed Student’s T tests.

### Extraction and analysis of metabolites

Soluble sugars (glucose, fructose and sucrose) were extracted from the same previously described leaf samples of five individual plants (the same four as in the RNA-seq plus one additional replicate) using ethanol and enzymatically quantified [133]. Total amino acids and nitrate were determined using the same extract, while starch and proteins were assayed in the insoluble material obtained after the ethanolic extraction, following the protocols described by Cross et al. [134]. Tre6P, phosphorylated intermediates and organic acids were extracted with chloroform/methanol, and measured by high-performance anion-exchange LC-MS/MS as described by Lunn et al. [135], with the modifications introduced by Figueroa et al. [33].

### Phylogenetic analysis

Protein sequences for TPS, SWEET, invertases and fructosyltransferases from *Triticum aestivum* (Ta), the progenitors of the A and D subgenomes *Triticum urartu* (Tu) and *Aegilops tauschii* (AEGt), *Triticum turgidum* (Tt), *Aegilops searsii* (As), *Oryza sativa* (Os), *Arabidopsis thaliana* (At), *Solanum lycopersicum* (Sl) and *Populus trichocarpa* (Pt) were downloaded from the NCBI database (https://www.ncbi.nlm.nih.gov/). Protein sequences from wheat were selected using the BLASTP algorithm in Ensembl Plants (http://plants.ensembl.org/index.html). Briefly, representative sequences from rice (for TPS, SWEET and invertases) or wheat (for fructosyltransferases) were used to search for homologs encoded in the *T. aestivum* genome (assembly TGACv1). The resulting sequences were downloaded from UniProt (http://www.uniprot.org/), manually curated and automatically aligned with those obtained for other species using the ClustalW program in GenomeNet (http://www.genome.jp/). Phylogenetic trees were built using the neighbor-joining algorithm (bootstrap of 1000) in Seaview 4.3.0 [136] and figures were prepared with the FigTree 1.3.1 program (http://tree.bio.ed.ac.uk/). Sequences used in this study are listed in Additional file 2: Table S4.

### Quantitative analysis of plant hormones

The endogenous phytohormones concentrations were determined in leaf samples by an ultra-high performance liquid chromatography–electrospray tandem mass spectrometry (UHPLC–MS/MS) using stable isotope-labelled internal standards as a reference. The isolation of CKs was performed according to Antoniadi et al. [137] and their levels were quantified by UHPLC–MS/MS [138]. Briefly, samples (20 mg FW) were extracted in 1 ml of modified Bieleski buffer [139] together with a cocktail of stable isotope-labeled internal standards used as a reference (0.25 pmol of CK bases, ribosides, *N*-glucosides, and 0.5 pmol of CK *O*-glucosides, nucleotides per sample added). The extracts were purified using the Oasis MCX column (30 mg/1 ml, Waters) and cytokinin levels were determined using the LC-MS/MS system consisting of an ACQUITY UPLC System and a Xevo TQ-S triple quadrupole mass spectrometer (Waters). The acidic phytohormones (JA; jasmonoyl-L-isoleucine, JA-Ile; *cis*-12-oxo-phytodienoic acid, *cis*-OPDA; indole-3-acetic acid, IAA; ABA; and SA) were extracted using an aqueous solution of methanol (10% MeOH/H_2_O, *v*/v) [140]. A cocktail of stable isotope-labelled standards was added as follows: 5 pmol of [^13^C_6_]IAA, 10 pmol of [^2^H_6_]JA, [^2^H_2_]JA-Ile, and [^2^H_6_]ABA, and 20 pmol of [^2^H_4_]SA and [^2^H_5_]OPDA (all from Olchemim Ltd., Czech Republic) per sample. The extracts were purified using Oasis HLB columns (30 mg/1 ml, Waters), targeted analytes were eluted using 80% MeOH and then analyzed by LC-MS/MS method [140]. The brassinosteroid content in the samples was determined using the previously described UHPLC–MS/MS method [141]. All experiments were performed in five independent biological replicates (the same four as in the RNA-seq plus one additional replicate).

## Additional files

Additional file 1: **Figure S1.** Representative pictures of MRCV symptoms in wheat at 21 dpi. Insets highlighting curled leaves and cross-cut edges are shown. **Figure S2.** Library mapping statistics. For each library, total reads, clean reads, mapped reads, and uniquely mapped reads that fall within a gene feature are indicated. T stands for treated (MRCV-infected) and C for control (mock-inoculated). R1 to R4 stand for each of the four plants sequenced from each treatment. **Figure S3.** qPCR validation of DATs at 21 dpi. For qPCR experiments, *n* = 6. Error bars: standard error. Statistical significance between RNA-seq and qPCR Fold Change values was assessed by Two-tailed Student’s T tests (*P < 0.05). **Figure S4.** Phylogenetic relationships of wheat vacuolar invertases and fructosyltransferases with other plant sequences. Protein sequences from *Triticum aestivum* (Ta), the progenitors of the A and D subgenomes *Triticum urartu* (Tu) and *Aegilops tauschii* (AEGt), *Triticum turgidum* (Tt), *Aegilops searsii* (As), *Oryza sativa* (Os), *Arabidopsis thaliana* (At), *Solanum lycopersicum* (Sl) and *Populus trichocarpa* (Pt) were used to build a phylogenetic tree using the neighbor-joining algorithm with a bootstrap of 1000. **Figure S5.** Phylogenetic relationships of wheat TPS with other plant TPS. Protein sequences of class II TPS from *Triticum aestivum* (Ta), *Oryza sativa* (Os), *Arabidopsis thaliana* (At), *Phaseolus vulgaris* (Pv) and *Populus trichocarpa* (Pt) were used to build a phylogenetic tree using the neighbor-joining algorithm with a bootstrap of 1000. **Figure S6.** Phylogenetic relationships of wheat SWEET with other plant SWEET. Sequences from *Triticum aestivum* (Ta), *Oryza sativa* (Os) and *Arabidopsis thaliana* (At) were used to build a phylogenetic tree using the neighbor-joining algorithm with a bootstrap of 1000. Wheat protein sequences whose transcripts were significantly increased or decreased in the RNA-seq analysis at 21 dpi are coloured in red and blue, respectively. (DOCX 2662 kb)

Additional file 2: **Table S1.** Differentially accumulated transcripts (DATs) upon MRCV infection at 12 and 21 dpi. For each gene, the normalized mean count for mock-inoculated and MRCV-infected samples, the fold change and the FDR-adjusted *P*-value (padj) are indicated. The annotations from Ensembl plants Release 37 or inferred in this work are included. **Table S2.** DATs classified as wheat Transcription factors according to reference [27]. **Table S3.** DATs classified as Carbohydrate-related according to their functional annotation. **Table S4.** Putative wheat invertases and fructosyltransferases, Tre6P synthases (TPS) and Sugars Will Eventually be Exported Transporters (SWEET) used to build the phylogenetic trees shown on Additional file 1: Figures S4-S6. Sequences encoded by DATs at 21 dpi are highlighted in red (increased upon the infection) and blue (decreased). A/N Inv, Vac Inv and CW Inv stand for acid and/or neutral, vacuolar and cell wall invertases respectively. Fructosyltransferases included sucrose:sucrose 1-fructosyltransferase (1-SST), fructan:fructan 1-fructosyltransferases (1-FFT) and sucrose:fructan 6-fructosyltransferases (6-SFT). **Table S5.** DATs classified as Amino acid-related according to their functional annotation. Amino acid transporters were extracted from reference [45]. **Table S6.** DATs classified as Hormone-related according to their functional annotation. **Table S7.** Primer sequences used for qRT-PCR validations. (XLSX 717 kb)

## Abbreviations

ABA: Abscisic acid
ARF: Auxin response factor
AUX: Auxin
BR: Brassinosteroid
cis-OPDA: Cis-12-oxo-phytodienoic acid
CK: Cytokinin
DAS-ELISA: Double Antibody Sandwich ELISA
DAT: Differentially accumulated transcript
dpi: Days post inoculation
ELISA: Enzyme-linked immunosorbent assay
ET: Ethylene
FDR: False discovery rate
GO: Gene ontology
IAA: Indole-3-acetic acid
JA: Jasmonic acid
JA-Ile: Jasmonoyl-L-isoleucine
LC-MS/MS: Liquid chromatography coupled to tandem mass spectrometry
MeJA: Methyl jasmonate
MRCV: Mal de Río Cuarto virus
PEP: Phosphoenolpyruvate
qRT-PCR: Quantitative reverse transcription PCR
RBSDV: Rice black streaked dwarf virus
SA: Salicylic acid
SL: Strigolactone
SRA: Sequence read archive
SWEET: Sugars Will Eventually be Exported Transporters
TCA: Tricarboxylic acid
TF: Transcription factor
TPS: Tre6P synthase
Tre6P: Trehalose 6 Phosphate
UHPLC–MS/MS: Ultra-high performance liquid chromatography coupled to tandem mass spectrometry
