# Supplementary material for: Mal de Río Cuarto virus infection causes hormone imbalance and sugar accumulation in wheat leaves
Source: BMC Plant Biol. 2019 Mar 22;19:112. doi: 10.1186/s12870-019-1709-y (PMC6431059; doi:10.1186/s12870-019-1709-y)
Supplement: Supplementary file 1 — Figure S1. Representative pictures of MRCV symptoms in wheat at 21 dpi. Insets highlighting curled leaves and cross-cut edges are shown. Figure S2. Library mapping statistics. For each library, total reads, clean reads, mapped reads, and uniquely mapped reads that fall within a gene feature are indicated. T stands for treated (MRCV-infected) and C for control (mock-inoculated). R1 to R4 stand for each of the four plants sequenced from each treatment. Figure S3. qPCR validation of DATs at 21 dpi. For qPCR experiments, n = 6. Error bars: standard error. Statistical significance between RNA-seq and qPCR Fold Change values was assessed by Two-tailed Student’s T tests (*P < 0.05). Figure S4. Phylogenetic relationships of wheat vacuolar invertases and fructosyltransferases with other plant sequences. Protein sequences from Triticum aestivum (Ta), the progenitors of the A and D subgenomes Triticum urartu (Tu) and Aegilops tauschii (AEGt), Triticum turgidum (Tt), Aegilops searsii (As), Oryza sativa (Os), Arabidopsis thaliana (At), Solanum lycopersicum (Sl) and Populus trichocarpa (Pt) were used to build a phylogenetic tree using the neighbor-joining algorithm with a bootstrap of 1000. Figure S5. Phylogenetic relationships of wheat TPS with other plant TPS. Protein sequences of class II TPS from Triticum aestivum (Ta), Oryza sativa (Os), Arabidopsis thaliana (At), Phaseolus vulgaris (Pv) and Populus trichocarpa (Pt) were used to build a phylogenetic tree using the neighbor-joining algorithm with a bootstrap of 1000. Figure S6. Phylogenetic relationships of wheat SWEET with other plant SWEET. Sequences from Triticum aestivum (Ta), Oryza sativa (Os) and Arabidopsis thaliana (At) were used to build a phylogenetic tree using the neighbor-joining algorithm with a bootstrap of 1000. Wheat protein sequences whose transcripts were significantly increased or decreased in the RNA-seq analysis at 21 dpi are coloured in red and blue, respectively. (DOCX 2662 kb) [file 12870_2019_1709_MOESM1_ESM.docx]

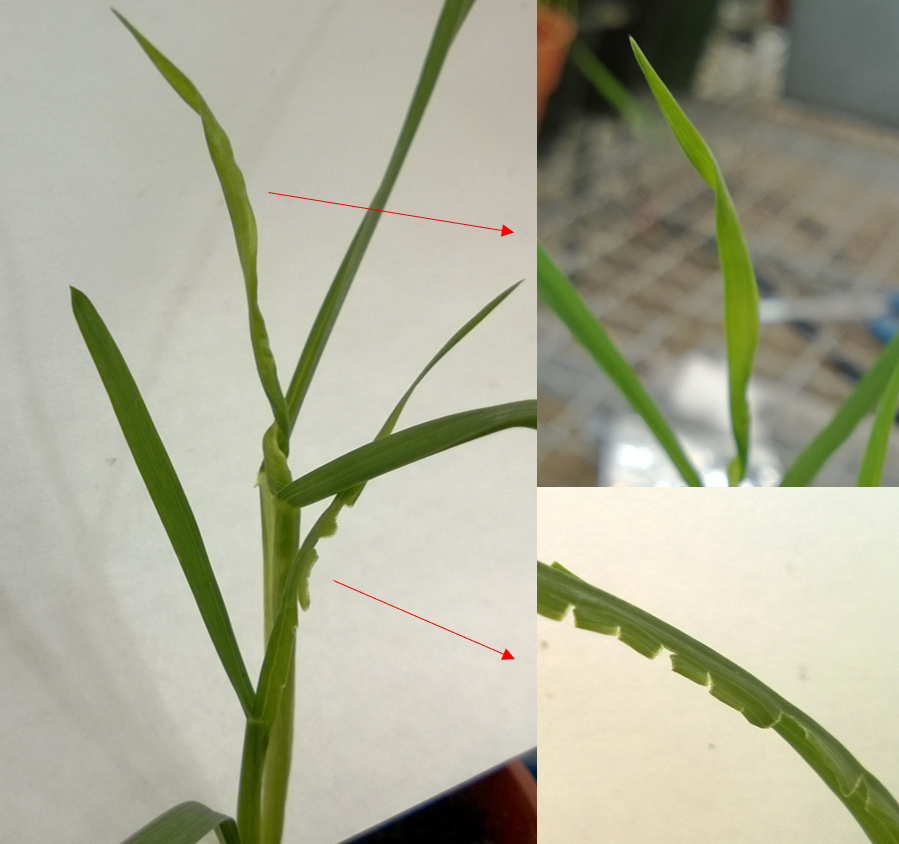


**Figure S1.** Representative pictures of MRCV symptoms in wheat at 21 dpi. Insets highlighting curled leaves and cross-cut edges are shown.

**Figure S1.** Representative pictures of MRCV symptoms in wheat at 21 dpi. Insets highlighting curled leaves and cross-cut edges are shown.


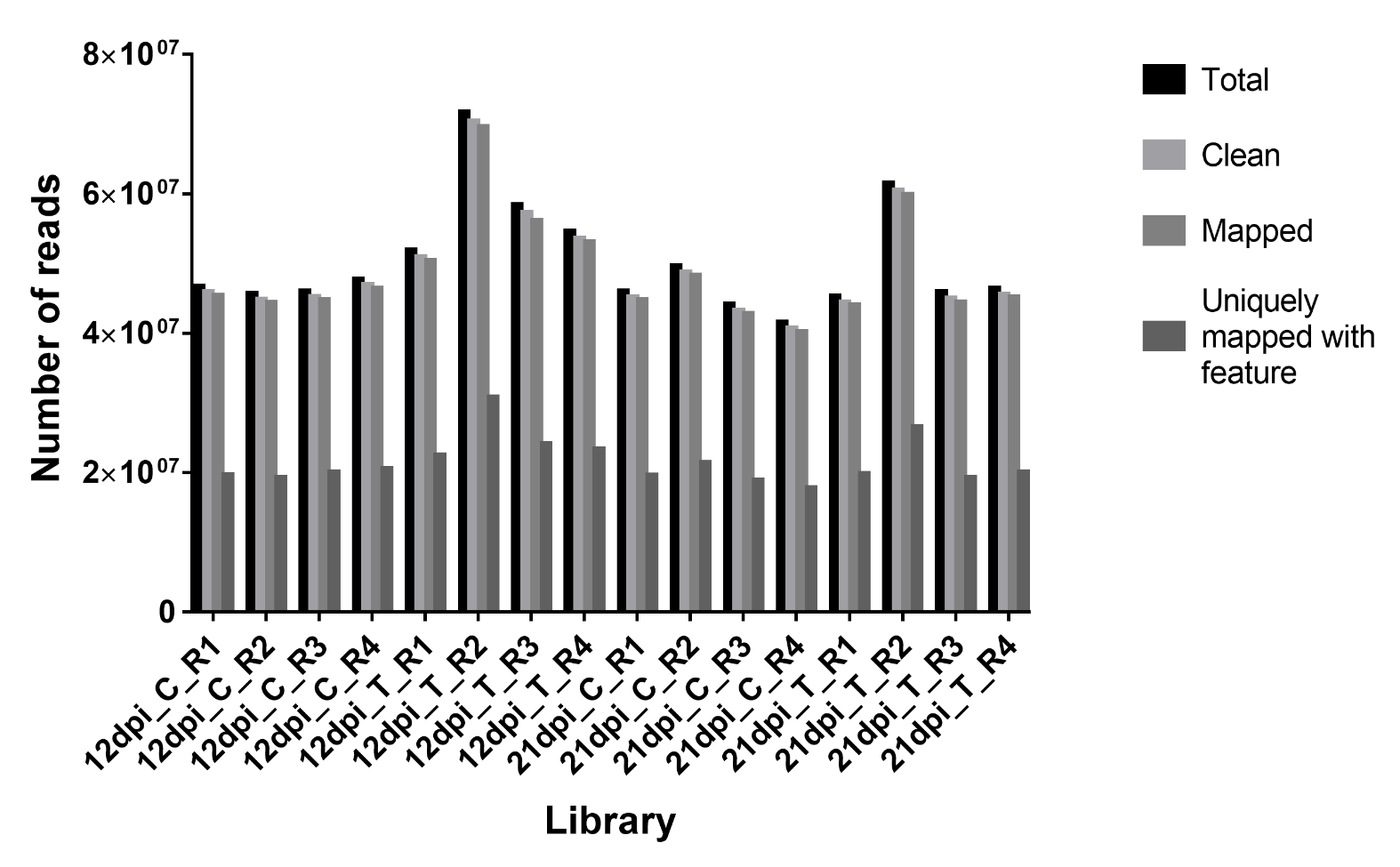


**Figure S2.** Library mapping statistics. For each library, total reads, clean reads, mapped reads, and uniquely mapped reads that fall within a gene feature are indicated. T stands for treated (MRCV-infected) and C for control (mock-inoculated). R1 to R4 stand for each of the four plants sequenced from each treatment.


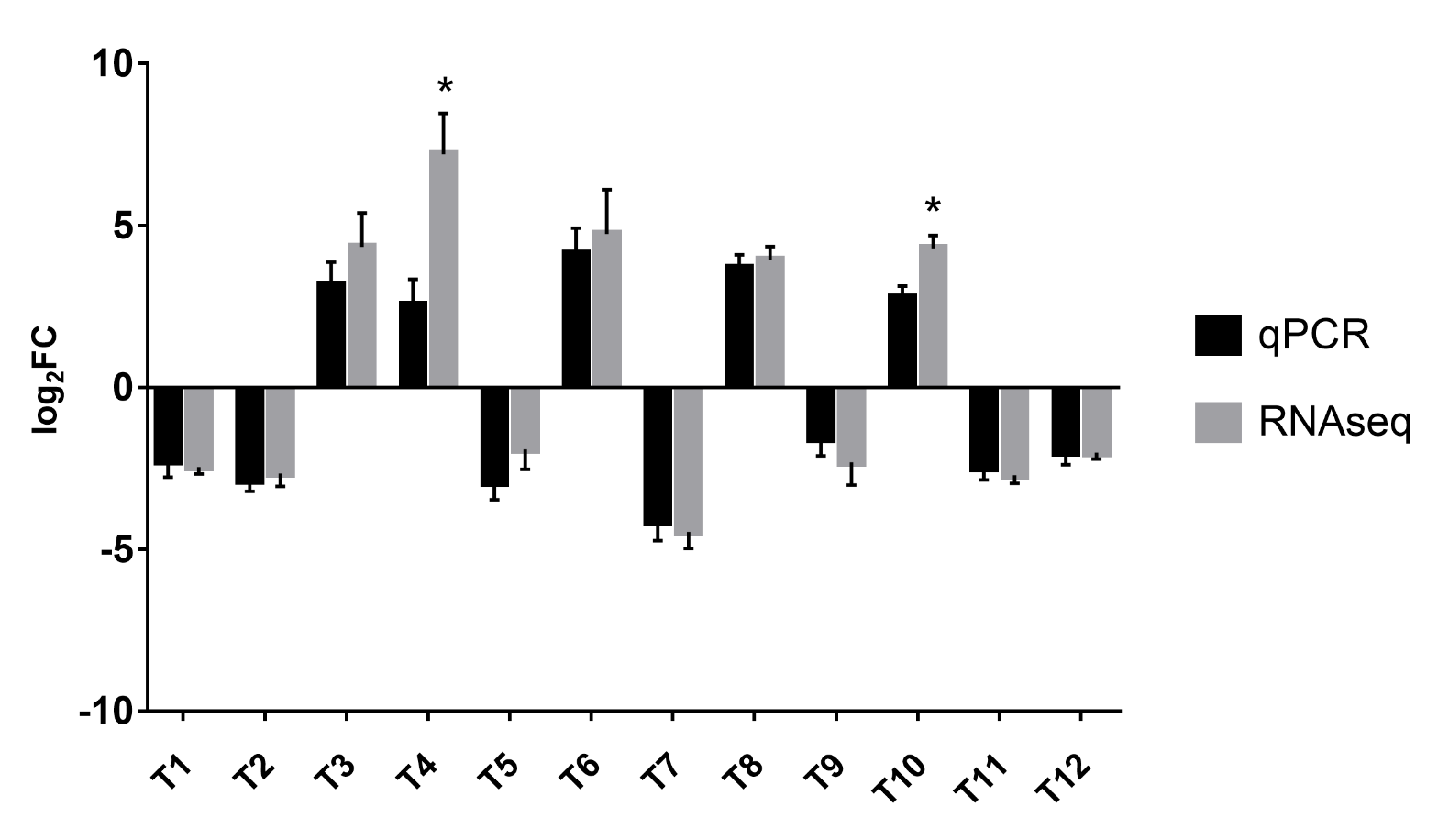


**Figure S3.** qPCR validation of DATs at 21 dpi. For qPCR experiments, n=6. Error bars: standard error. Statistical significance between RNA-seq and qPCR Fold Change values was assessed by Two-tailed Student's T tests (**P*<0.05). Transcript (T) 1 to 12 are TRIAE_CS42_2BL_TGACv1_130477_AA0412140, TRIAE_CS42_2DL_TGACv1_158627_AA0523580, TRIAE_CS42_4AL_TGACv1_290986_AA0991190, TRIAE_CS42_4DL_TGACv1_342936_AA1125650, TRIAE_CS42_5AL_TGACv1_374149_AA1191800, TRIAE_CS42_5BL_TGACv1_405628_AA1331730, TRIAE_CS42_5BS_TGACv1_423880_AA1384600, TRIAE_CS42_5DS_TGACv1_457428_AA1486420, TRIAE_CS42_6DL_TGACv1_528006_AA1710710, TRIAE_CS42_7AS_TGACv1_570678_AA1839270, TRIAE_CS42_7BS_TGACv1_594019_AA1955580, and TRIAE_CS42_7DS_TGACv1_622233_AA2035940 respectively.


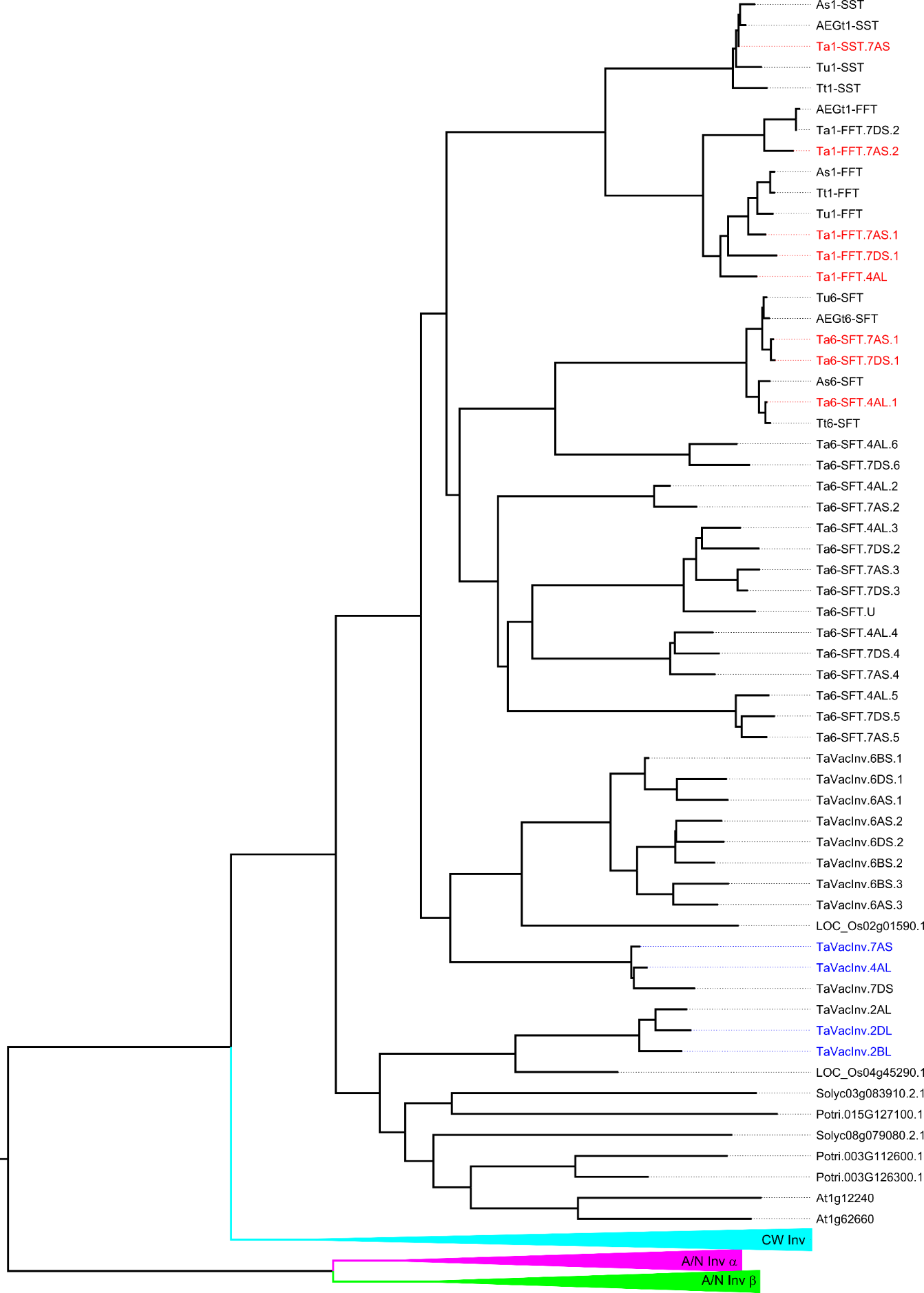


**Figure S4.** Phylogenetic relationships of wheat vacuolar invertases and fructosyltransferases with other plant sequences. Protein sequences from *Triticum aestivum* (Ta), the progenitors of the A and D subgenomes *Triticum urartu* (Tu) and *Aegilops tauschii* (AEGt), *Triticum turgidum* (Tt), *Aegilops searsii* (As), *Oryza sativa* (Os), *Arabidopsis thaliana* (At), *Solanum lycopersicum* (Sl) and *Populus trichocarpa* (Pt) were used to build a phylogenetic tree using the neighbor-joining algorithm with a bootstrap of 1000. The percentage of replicate trees in which the associated taxa clustered together in the bootstrap test is shown next to the branches. Wheat protein sequences whose transcripts were significantly increased or decreased in the RNA-seq analysis at 21 dpi are coloured in red and blue, respectively. All the sequences used and their references are listed on **Additional file 2: Table S3**. Acid and/or neutral (A/N) and cell wall (CW) invertases clades were collapsed for simplicity.


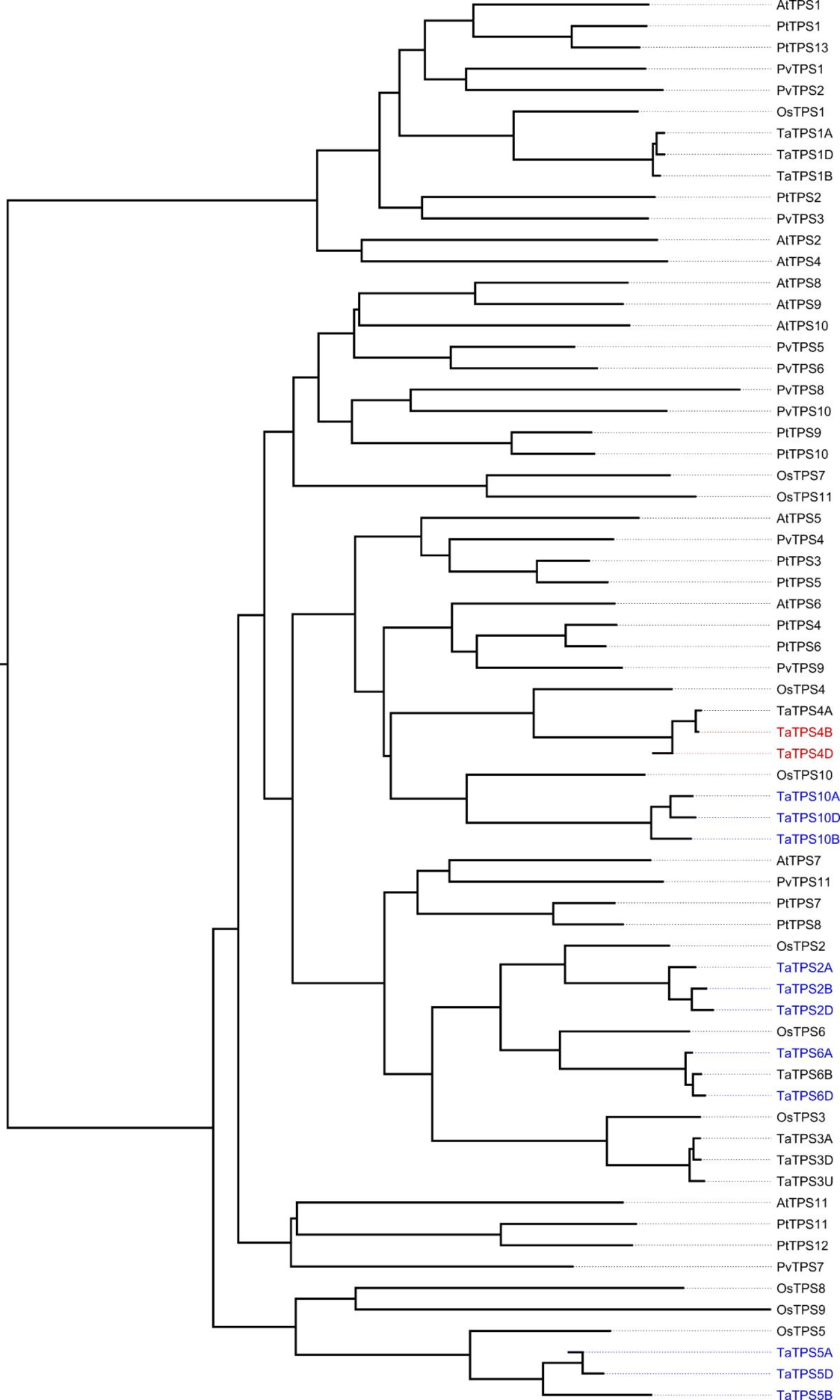


**Figure S5.** Phylogenetic relationships of wheat TPS with other plant TPS. Protein sequences of class II TPS from *Triticum aestivum* (Ta), *Oryza sativa* (Os), *Arabidopsis thaliana* (At), *Phaseolus vulgaris* (Pv) and *Populus trichocarpa* (Pt) were used to build a phylogenetic tree using the neighbor-joining algorithm with a bootstrap of 1000. The percentage of replicate trees in which the associated taxa clustered together in the bootstrap test is shown next to the branches. Wheat protein sequences whose transcripts were significantly increased or decreased in the RNA-seq analysis at 21 dpi are coloured in red and blue, respectively. All the sequences used and their references are listed on **Additional file 2: Table S3**. The class I TPS clade was collapsed for simplicity.


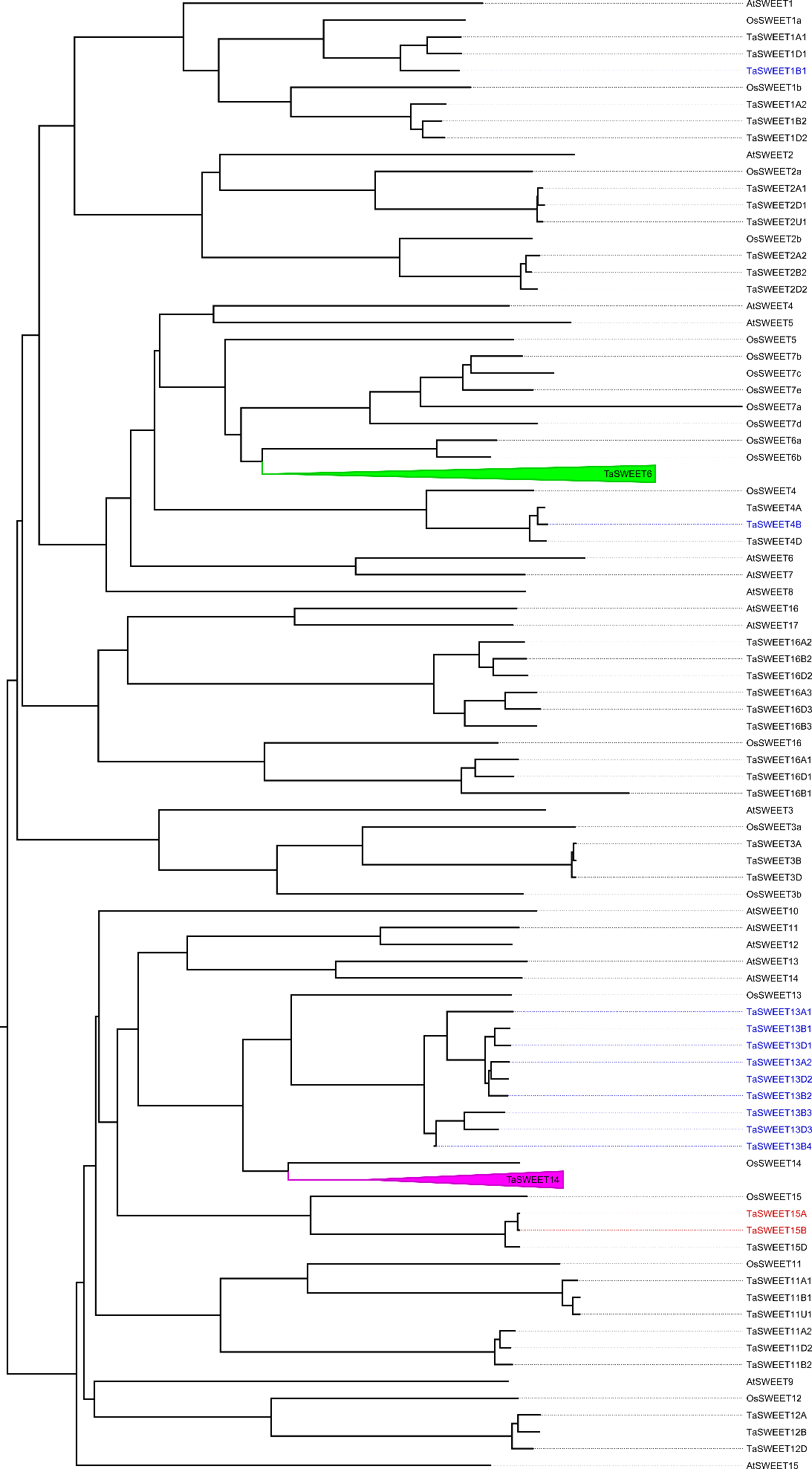


**Figure S6.** Phylogenetic relationships of wheat SWEET with other plant SWEET. Sequences from *Triticum aestivum* (Ta), *Oryza sativa* (Os) and *Arabidopsis thaliana* (At) were used to build a phylogenetic tree using the neighbor-joining algorithm with a bootstrap of 1000. The percentage of replicate trees in which the associated taxa clustered together in the bootstrap test is shown next to the branches. Wheat protein sequences whose transcripts were significantly increased or decreased in the RNA-seq analysis at 21 dpi are coloured in red and blue, respectively. All the sequences used and their references are listed on **Additional file 2: Table S3**. SWEET 2, 6, 16/17, 14 and 11 sequences were collapsed for simplicity.
